# Amniotic Epithelial Cells: A New Tool to Combat Aging and Age-Related Diseases?

**DOI:** 10.3389/fcell.2016.00135

**Published:** 2016-11-22

**Authors:** Clara Di Germanio, Michel Bernier, Rafael de Cabo, Barbara Barboni

**Affiliations:** ^1^Faculty of Veterinary Medicine, University of TeramoTeramo, Italy; ^2^Translational Gerontology Branch, National Institute on Aging, National Institute of HealthBaltimore, MD, USA

**Keywords:** amniotic cells, aging, inflammation, tumorigenesis, regenerative medicine, rejuvenation

## Abstract

The number of elderly people is growing at an unprecedented rate and this increase of the aging population is expected to have a direct impact on the incidence of age-related diseases and healthcare-associated costs. Thus, it is imperative that new tools are developed to fight and slow age-related diseases. Regenerative medicine is a promising strategy for the maintenance of health and function late in life; however, stem cell-based therapies face several challenges including rejection and tumor transformation. As an alternative, the placenta offers an extraordinary source of fetal stem cells, including the amniotic epithelial cells (AECs), which retain some of the characteristics of embryonic stem cells, but show low immunogenicity, together with immunomodulatory and anti-inflammatory activities. Because of these characteristics, AECs have been widely utilized in regenerative medicine. This perspective highlights different mechanisms triggered by transplanted AECs that could be potentially useful for anti-aging therapies, which include: Graft and differentiation for tissue regeneration in age-related settings, anti-inflammatory behavior to combat “inflammaging,” anti-tumor activity, direct lifespan and healthspan extension properties, and possibly rejuvenation in a manner reminiscent of heterochronic parabiosis. Here, we critically discuss benefits and limitation of AECs-based therapies in age-related diseases.

## Introduction

Among the health challenges of this century, aging itself represents one of the main risk factor for diseases (Niccoli and Partridge, 2012). In fact, aging is associated with a general decline in the homeostasis and regeneration of the body, which often leads to common age-related diseases. The mechanisms underlying age-related dysfunctions are heterogeneous and include a generalized decrease in tissue regenerative responses (López-Otín et al., 2013). In the adult body, tissue-specific stem cells are involved in the regeneration and repair of various tissues and organs, and, for a long time, they were considered as a fountain of youth and a panacea to cure several diseases. Fetal stem cells are gaining great interest in regenerative medicine, especially in the last decades, due to their ease of collection and safety (Miki et al., 2005). A large amount of preclinical studies has confirmed that fetal stem cells are the ideal candidates for transplantation and organ repair (Silini et al., 2015) because they are non-tumorigenic after transplantation (Akle et al., 1981). Furthermore, the immunomodulatory properties of amniotic membrane-derived stem cells allow their use in anti-inflammatory therapies (Insausti et al., 2014). Transplantation of amniotic epithelial cells (AECs) has already been tested for different diseases in preclinical settings; however, more recently, a few studies have also tested their potential in fighting age-associated disorders in animal models.

In this perspective, we will discuss all these aspects with a particular focus on how aging research could potentially benefit from the utilization of AECs.

## Stem cells sources

**Figure 1:**
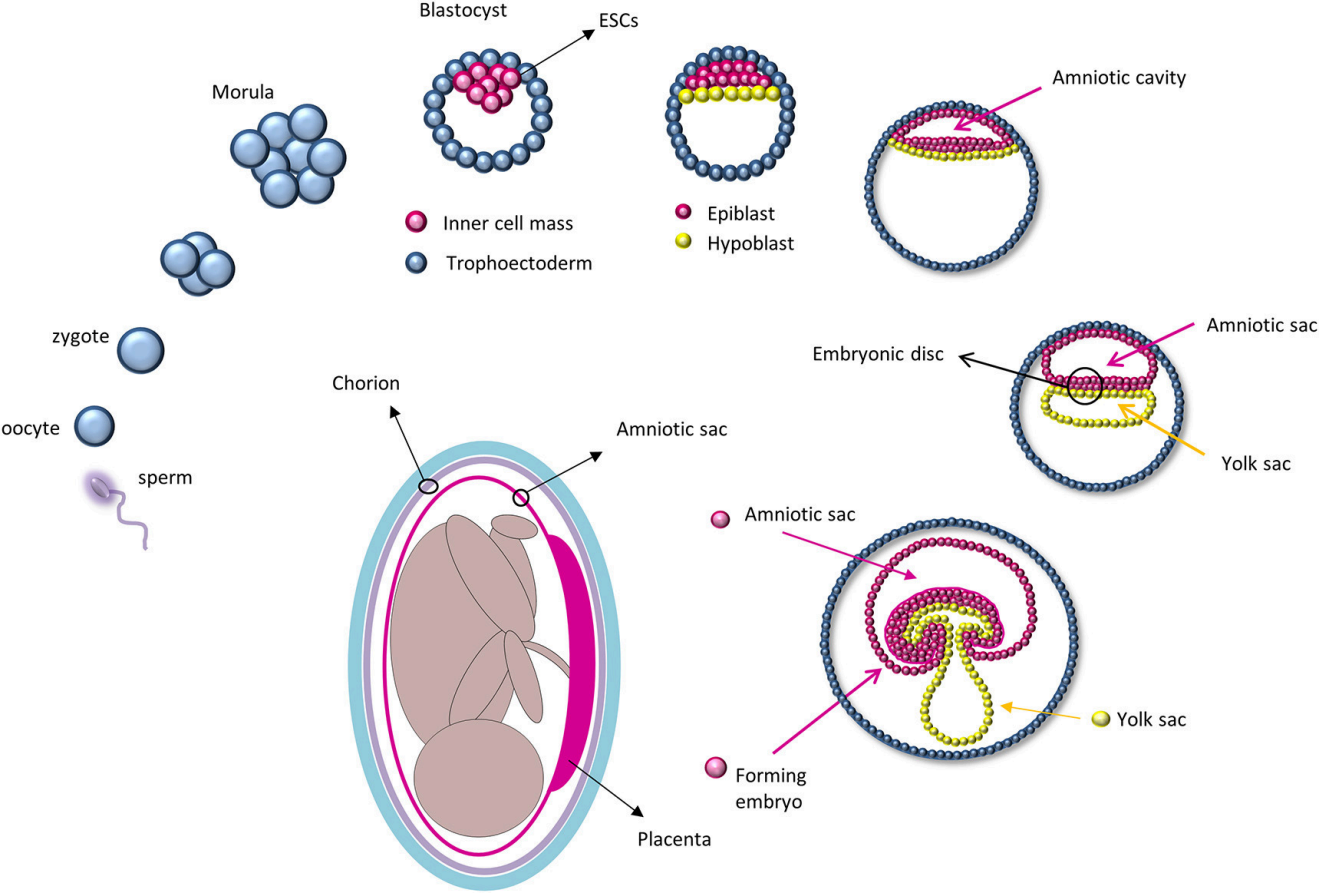
**Development of the amniotic sac during embryogenesis: After fertilization, the zygote undergoes a series of cell division and starts to differentiate at the blastocyst stage in which only cells of the inner cell mass, and subsequently the epiblast, will give rise to the embryo**. Amniotic epithelial cells are collected from the amniotic sac that will form directly from the epiblast. (The path from the epiblast to the amniotic cells is depicted in magenta).

Stem cells are specialized cells of the body that are capable of self-renewal and differentiation. For regenerative therapy, various cell populations at different developmental stages have been considered for transplantation, including embryonic stem cells (ESCs), fetal and adult cells, and experimentally generated induced pluripotent stem cells (iPSC). The zygote is perhaps the most primitive “stem cell” and, right after fertilization, it undergoes a series of rapid divisions that all appear to be self-renewal divisions as every single cell of the blastomere is undifferentiated. From this state, differentiation divisions begin to occur as cells rearrange themselves at day 5 to form a cavity (blastocoele) in which an inner cell mass is surrounded by trophoectoderm cells (Moore and Persaud, 1998; Figure 1). It is at this stage that ESCs are collected from the inner cell mass. Because they retain pluripotency features (Thomson et al., 1998), ESCs are considered the gold standard of stem cells: They are widely used for basic research to study *in vivo* embryogenesis and development and for regenerative medicine (Murry and Keller, 2008), although with some limited success. In fact, ESCs are highly pluripotent cells capable of forming teratomas after transplantation and may be subject to rejection. The ethical concerns regarding the collection of ESCs from embryos has led researchers to overcome these limitations by using iPSCs, adult cells reprogrammed to a pluripotency state by forced expression of specific genes. In 2006, Yamanaka and colleagues were the first to describe that the introduction of four genes encoding the transcription factors *Oct4* (octamer-binding protein 4, also known as Pou5f1), *Sox2* (SRY-related HMG-box gene 2), *cMyc*, and *Klf4* (Kruppel-like factor 4) could convert adult cells into pluripotent stem cells (Takahashi and Yamanaka, 2006). This represented a milestone in stem cell biology as it opened new approaches to study and combat diseases. Unfortunately, forced expression of *cMyc* and *Klf4*, which encode proto-oncogene proteins, increases the rate of cancer transformation (Ben-David and Benvenisty, 2011). More recently, studies have used other genes and different techniques to improve the poor efficiency of iPSC reprogramming; nevertheless, these cells still exhibit tumorigenic properties *in vivo* and can elicit an immune response (Zhao et al., 2011).

Adult tissue specific stem cells, like hematopoietic precursors, muscle satellite cells, and bone marrow-derived mesenchymal stem cells, are also limited in their potential for regenerative medicine as their function is limited to only their specific tissue, they can induce immune-rejection responses, and acquiring adequate numbers of tissue-specific stem cells for regenerative studies can often be challenging due to their rarity as well as limited *ex vivo* maintenance and expansion techniques (Müller et al., 2016).

A potential alternative to circumvent these limitations that emerged in the last decades is the utilization of fetal-derived stem cells. Amniotic stem cells can be collected from different fetal annexes (amnion, chorion, amniotic fluid, Wharton jelly) and have been proven to be safe, easy to collect, and devoid of immunogenic and tumorigenic properties (Mamede et al., 2012; Saito et al., 2012; Murphy and Atala, 2013; Pozzobon et al., 2014). AECs are collected from the epithelial layer of the amnion which derives directly from the epiblast as it retains some ESC characteristics. In fact, after implantation, the inner cell mass re-organizes and, driven by differential gene expression, divides into a double layer of cells (Zernicka-Goetz et al., 2009), i.e., the hypoblast that migrates to the free surface of the inner cell mass and gives rise to the endoderm; and the epiblast, which will form the rest of the embryo (Zernicka-Goetz et al., 2009). Before gastrulation, cells from the epiblast form a membrane, the amnion, within which the human embryo and later the fetus develops until birth (Moore and Persaud, 1998; Figure 1). AECs express some of the ESCs surface markers, such as stage-specific embryonic antigens (SSEA) 3 and 4, tumor rejection antigens (TRA) 1-60 and 1-81, and molecular markers of pluripotency, including Oct4, Sox2, and Nanog (Parolini et al., 2008). Their plasticity was first confirmed *in vitro* by chimera formation using mouse ESCs (Tamagawa et al., 2004) and later by the production of cell types from all three germ layers using specific cell differentiation protocols (Miki et al., 2005; Parolini et al., 2008).

## AECs transplantation to improve organ functions

**Figure 2:**
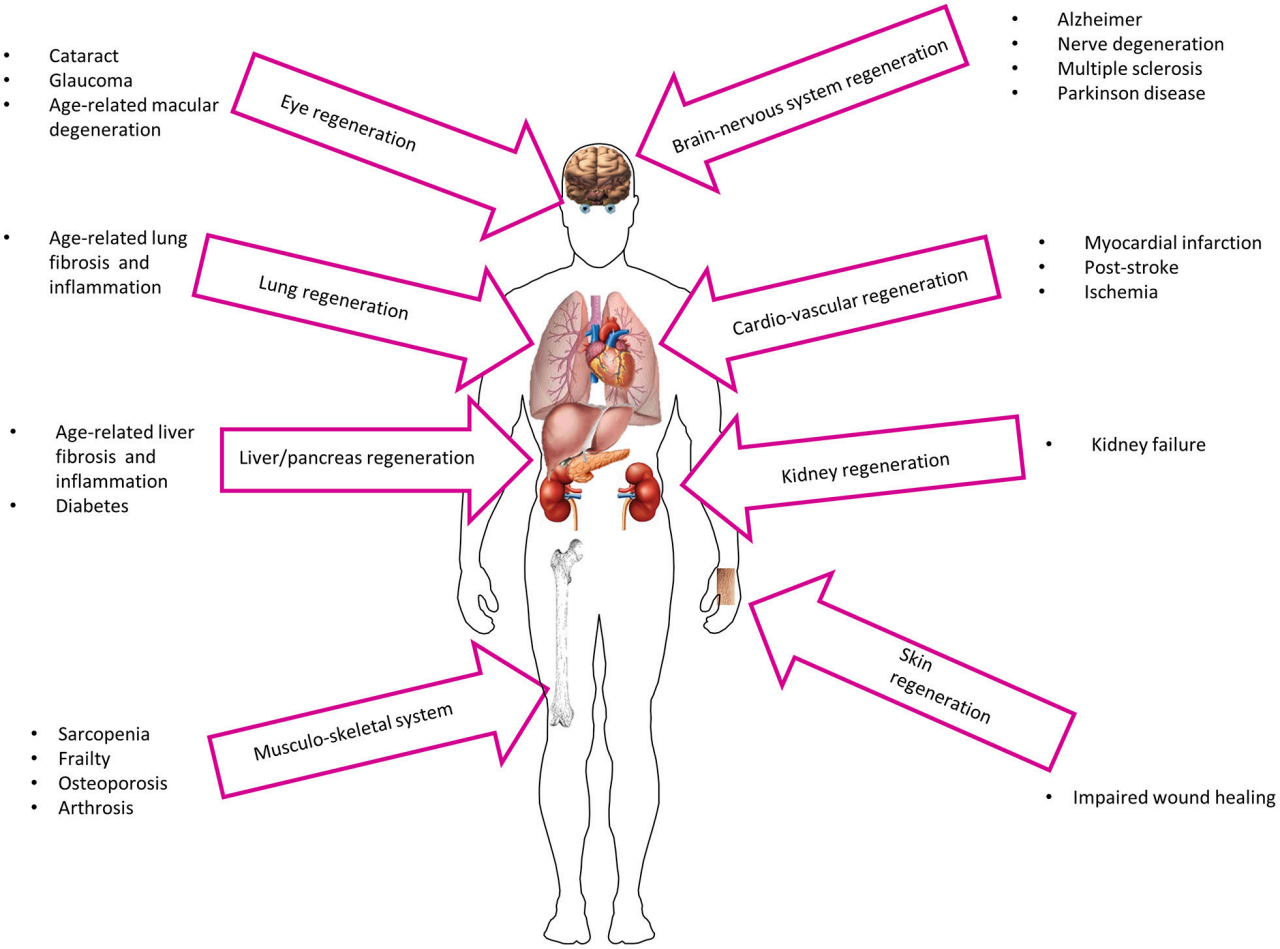
**Potential utilization of AECs in age-related diseases**.

With old age, a generalized malfunction of stem cells may be responsible for the emergence of various chronic diseases, such as osteoporosis, type 2 diabetes mellitus, Parkinson's disease, atherosclerosis, and cancer, to name a few (Boyette and Tuan, 2014). Although the integrity of aging tissues is severely compromised by the altered number and function of stem cells, the reactivation of specific populations of progenitor cells through cell transplantation may represent a viable avenue in anti-aging therapies (Figure 2). Since the beginning of the twentieth century, amniotic cells have been shown to have potential in regenerative medicine (Silini et al., 2015), with transplanted AECs implementing the healing process either through direct grafting of the tissue/organ to be repaired or by the secretion of anti-inflammatory and anti-fibrotic molecules (discussed below), although most of the times both mechanisms act simultaneously.

More recently, several findings in preclinical research have shown successful grafting and active proliferation of AECs when transplanted in injured organs of various animal models, which led to the establishment of a dozen clinical studies (www.clinicaltrials.gov). The beneficial effects of amniotic cell therapy have been shown also in several tissues that are implicated in age-related diseases, including the nervous system/brain and liver as well as the cardiovascular and musculoskeletal systems (Figure 2). For example, in rat models, AECs can graft in the nerve fiber in the corpus callosum in healthy animals (Wu et al., 2012) and participate in the regeneration of transected optic nerve to facilitate the process of neural restoration following injury (Xie et al., 2015a). The therapeutic potential of AECs is further illustrated by their ability to repair and/or improve brachial plexus injury (Yang et al., 2014) and spinal cord injury (Meng et al., 2008), while improving markers of Parkinson's disease in rats (Yang et al., 2010). Moreover, mechanical allodynia caused by microglia activity in injured spinal cord is significantly suppressed with AECs transplantation (Roh et al., 2013). Similarly, AECs can reduce brain edema and motor deficit in intracerebral hemorrhagic rats (Dong et al., 2010). The transplantation of human AECs mitigates many age-associated phenotypes in the Niemann-Pick type C1 mice including extension of life span, reduction in rapid weight loss, and alleviation of tissue damage (Hong et al., 2012).

Grafting of AECs in the liver of immunocompromised SCID mice promotes their differentiation into hepatocytes (Miki et al., 2009; Marongiu et al., 2011) while injection of AECs around the cardiovascular infarction area enables their differentiation into cardiomyocyte-like cells with ensuing decrease in infarct size and improved cardiac function in rats (Fang et al., 2012). Other therapeutic applications of AECs in laboratory animals include support of the regenerative process in tendon defects (Barboni et al., 2012a,b) via collagen type I production (Muttini et al., 2013), improvement in bone regeneration *in vivo* through implantation of scaffold-loaded AECs (Barboni et al., 2013), and reduction in the early host immune response to the scaffold (Jiawen et al., 2014).

Despite these advances, there are some limitations including the fact that the large majority of the studies were conducted in young animal recipients and, therefore, the efficacy of AECs transplantation in humans remains largely untested. This is especially true for elderly patients due to the bigger risk of graft failure (Hubbard and Dashti, 2011); indeed, challenges in transplantation are greater in older adults than in younger recipients, partly due to underlying pathologies, dysregulation of the immune system, and uncertain long-term outcomes after organ transplantation (Goldstein, 2012). Nevertheless, the results in preclinical research appear promising in the treatment and cure for age-related conditions and deserve further investigation.

## Combat inflammaging

Persistent low grade inflammation accelerates the aging process and is involved in multiple diseases. This process, termed “inflammaging,” has been associated with tissue degeneration and increased mortality and morbidity in the elderly (Franceschi et al., 2000). Intrinsic and extrinsic factors contribute to inflammaging, which include immunosenescence and/or abnormal extrinsic activation of the immune system either by environmental factors, defective somatic cells, or senescent cells (Franceschi and Campisi, 2014). Senescence is a protective mechanism that cells mount in response to different stress stimuli to avoid possible malignant transformation (Rodier and Campisi, 2011). Senescent cells cease to proliferate, but they are metabolically and transcriptionally active, capable of releasing inflammatory mediators implicated in the senescence-associated secretory phenotype (SASP) (Coppé et al., 2010). The secretion of IL-6, IL1β, and TNFα during inflammaging is a consequence of the activation of NF-κB and STAT transcription factors (Bollrath and Greten, 2009), and is involved in the pathogenesis of most age-associated diseases. In fact, the levels of these cytokines are usually significantly higher in elderly patients with cancer, cardiovascular diseases, rheumatoid arthritis, diabetes, multiple sclerosis, and cognitive decline than in healthy individuals (Prasad et al., 2012). It follows that the potential anti-inflammatory properties of transplanted amniotic cells could be used as a therapy to combat inflammaging.

The immune-privileged nature of placenta-derived AECs allows maternal tolerance of the fetus. During pregnancy, the mother's body hosts a semi-allogenic fetus and has developed subtle tolerance mechanisms to avoid rejection due to paternal antigens and impairment in the general response to pathogens (Hemberger, 2013). Subpopulations of leukocytes of the innate and adaptive immune systems that reside in the placenta increase during pregnancy (Hemberger, 2013), consistent with a transient, antigen-specific immune suppression. The low levels of both MHC class I surface antigens and the major components of the antigen-processing machinery represent a highly conserved feature of AECs among various animal species (Banas et al., 2008) despite differences in embryogenesis and formation of the annexes. AECs constitutively express the non-classical human leukocyte antigen G (HLA-G) (Insausti et al., 2014), this is tissue-restricted and is usually found in immune-privileged organs like eye, testis and brain, where it exerts tolerogenic properties by modulating the activities of both T and B lymphocytes, NK cells, and dendritic cells (Rouas-Freiss et al., 1999).

Due to these unique characteristics, AECs are a valuable tool for fighting inflammaging. The mechanisms implicated in the immunosuppressive and immunomodulatory properties of AECs have been elucidated using various target cells of the innate and the adaptive immune systems. AECs inhibit alloreactive T-lymphocytes in a mixed lymphocyte reaction (Pianta et al., 2015) and they also foster the switch from T-helper (Th) 1 and Th17 lymphocytes, to Th2 and Treg (Pianta et al., 2015), and M1 to M2 macrophages (Manuelpillai et al., 2012) through the release of anti-inflammatory cytokines. AECs also block maturation of monocytes into dendritic cells (Magatti et al., 2009) and inhibit proliferation of activated peripheral blood mononuclear cells *in vitro* (Wolbank et al., 2007) partly through the secretion of anti-inflammatory cytokines (Kang J. W. et al., 2012).

AECs reduce pulmonary fibrosis and inflammation (Moodley et al., 2010) through Treg cell regulation (Tan et al., 2015). AECs have been found to also lower the number of pulmonary leucocytes and expression of pro-inflammatory markers (e.g., TGF-β, PDGF-α, PDGF-β, TNF-α, IFN-γ, and IL-6) that resulted in the normalization of lung tissue density, collagen content, and production of smooth muscle actin (α-SMA) (Murphy et al., 2011; Vosdoganes et al., 2013a). Moreover, AECs promote a pro-reparative M2 phenotype by directly modulating macrophage behavior (Tan et al., 2014). When administered to neonatal mice, AECs partially reduce hyperoxia-induced inflammation and structural damage of the lung (Vosdoganes et al., 2013b) while attenuating the fetal pulmonary inflammatory response to experimental intrauterine inflammation in sheep (Vosdoganes et al., 2011).

AEC transplantation also results in an effective treatment for liver inflammation and fibrosis. Sustained hepatic inflammation is a key prerequisite for fibrogenesis and is characterized by an activation of fibrogenic hepatic stellate cells and macrophages, both of which being reverted by the treatment with AECs (Manuelpillai et al., 2012; Hodge et al., 2014). The anti-fibrotic activity of transplanted AECs is accompanied by a reduction in hepatic TNF-α, IL-6, and TGF-β levels, and an induction in IL-10 levels and metalloproteinase-2 activity (Manuelpillai et al., 2010). Studies on the nervous system have also reported that AECs inhibit microglia activation, reduce brain edema, and improve neurological deficit in a rat model of intracerebral hemorrhage through downregulation of TNF-α, Il-1β, and MMP-12 (Liang et al., 2014). The transplantation of AECs may even be beneficial against multiple sclerosis (Liu et al., 2012, 2014) and in immunosuppressant therapy with implication in pancreatic islet transplantation (Qureshi et al., 2011).

## Anti-tumor effects of AECs

Carcinogenesis is one of the leading causes of death in the older population. The utilization of stem cell transplantation is now being considered an anticancer therapy along with surgery, chemotherapy, and radiotherapy. Non-engineered and engineered stem cells have a number of features that make them useful as gene delivery vehicles in anticancer protocols (Kang et al., 2012a). In a breast cancer model, AECs reduce tumor progression and tumor size without compromising the normal breast tissue (Kang et al., 2012b). There is evidence that AECs inhibit tumor growth through the release of soluble molecules responsible for cell cycle blockade and/or apoptosis induction. Injection of AECs into growing tumors reduces their final size through increased activation of a proapoptotic signaling response (e.g., caspase 3 and 8 activity) and reduction in Bcl-2 protein levels (Niknejad et al., 2014). Finally, AEC transplantation blocks tumor proliferation in the G0/G1 phase by inhibiting cyclins (D2, E1, and H) and cyclin-dependent kinases without induction of apoptosis (Magatti et al., 2012; Di Germanio et al., 2016).

The notion that AECs possess anti-angiogenic properties that would inhibit or at least decrease nutrient and oxygen availability to combat metastasis spread of malignant tumors is rather controversial (Zhu et al., 2016). It is likely that the conflicting conclusions reached on the anti- vs. pro-angiogenic properties of AECs depend on the different tissues and/or experimental outcomes measured, ranging from tumor growth to epithelialization.

## Direct anti-aging and rejuvenating effects

The utilization of AECs in regenerative medicine already covers a vast array of diseases and can ultimately be of use in anti-aging therapies. The transplantation of amniotic cells in a mouse model of premature aging delays senescence in the recipient animals following grafting and differentiation into multiple tissues and organs (Xie et al., 2015b). Lymphocyte dysmaturity and osteoporosis are also ameliorated after transplantation of amniotic cells into Bmi-1^−/−^ mice, with a subsequent decrease in oxidative stress and DNA damage (Xie et al., 2015b). Once-a-month transplantation of amniotic cells slows cognitive decline and improves the physical function of aging rats, leading to an increase in lifespan (Kim et al., 2015). Similarly, it was previously shown that transplantation of young mesenchymal stromal cells significantly slows the loss of bone density and prolongs the lifespan of old mice (Shen et al., 2011). We recently reported that the blockade of SASP by AEC-derived conditioned medium delays the onset of senescence in cultured fibroblasts (Di Germanio et al., 2016), consistent with the idea that extrinsic environmental factors can influence cell behavior. Vital organs, tissues, and the *in vivo* milieu slowly loose some function during aging. The technique of heterochronic parabiosis, where two animals of different ages are joined together surgically to create shared circulatory system, illustrates that factors in young blood can rejuvenate aged stem cells from muscle, liver and brain in mice (Conboy et al., 2005). The administration of young blood improves also cognitive function and rejuvenates synaptic plasticity in old mice (Villeda et al., 2014), supporting the notion that cellular aging not only depends on intrinsic factors, such as DNA damage and ROS accumulation, but also relies on extrinsic factors (Conboy et al., 2015). To date, GDF11 (although very controversial) (Loffredo et al., 2013), oxytocin (Elabd et al., 2014), klotho (Kuro-o et al., 1997) have been described as “rejuvenation” circulating factors and it remains to be seen whether they can provide a youthful phenotype in the entire aged animal.

Pregnancy represents the only naturally occurring heterochronic parabiosis system, where fetal and maternal entities coexist and share the same circulation. A small number of fetal cells appears in the circulatory system and organs of the mother, starting at 6 weeks of pregnancy (Bianchi, 2012; Nelson, 2012), and this fetal microchimerism can somehow rejuvenate the mother (Falick Michaeli et al., 2015a). Recruitment of fetal progenitor cells to maternal wounds has been found to participate in the inflammatory and angiogenic responses (Nassar et al., 2012a,b; Mahmood and O'Donoghue, 2014). In addition, pregnancy offers beneficial effect on liver and muscle regeneration even though no fetal cells have been found at the site of injury (Gielchinsky et al., 2010; Falick Michaeli et al., 2015b). However, microchimerism can also elicit detrimental effects, especially in auto-immune diseases (Nelson, 2012). The origin of the cells involved in microchimerism is unknown, but amniotic cells have been proposed to be implicated in this phenomenon (Rosner and Hengstschläger, 2013). From this body of data, one could speculate that the presence of very young amniotic cells in the mother's circulation exerts similar effects and, therefore, can explain the beneficial and rejuvenating properties of amniotic cell transplantation.

## Conclusions and current limitations

Regenerative medicine strategies to combat age-associated phenotypes will benefit substantially from improved understanding of the intricate biology of stem cells. Following transplantation, exogenous stem cells can graft and replace dysfunctional tissues or improve the tissue milieu, possibly through anti-inflammatory and anti-fibrotic mechanisms. Moreover, the AEC secretome contains an array of molecules and proteins capable of replicating most of the properties of AECs (Di Germanio et al., 2016). Although these strategies may ultimately help alleviate some age-related diseases, there are some important caveats that limit the translation of animal studies into clinical trials. For example, although these cells can be expanded and maintained *ex vivo*, the yield and recovery of the number of AECs can be quite variable and their characteristics appear to be dependent on the genotype and age of the donor and the collection method. Furthermore, the large majority of existing studies on transplantation have been conducted on young rather than old animals and, therefore, these findings need to be independently confirmed.

In conclusion, there is still a long road ahead before stem cell-based therapies can be used in clinical settings; however, existing animal studies are encouraging and offer the opportunity for the development and implementation of exploratory clinical trials aimed at providing promising new therapeutic avenues in the fight against age-related diseases.

## Author contributions

CD: conception and design, manuscript writing, figures drawing. MB: manuscript revision and editing, final approval of manuscript. RD and BB: Financial support, revision and final approval of manuscript.

### Conflict of interest statement

The authors declare that the research was conducted in the absence of any commercial or financial relationships that could be construed as a potential conflict of interest.
